# Combined ocular and cervical vestibular evoked myogenic potential in individuals with vestibular hyporeflexia and in patients with Ménière's disease^☆^^☆☆^

**DOI:** 10.1016/j.bjorl.2016.04.017

**Published:** 2016-05-31

**Authors:** Tatiana Rocha Silva, Luciana Macedo de Resende, Marco Aurélio Rocha Santos

**Affiliations:** a Universidade Federal de Minas Gerais (UFMG), Faculdade de Medicina, Programa de Pós-graduação em Ciências Fonoaudiológicas, Belo Horizonte, MG, Brazil; b Universidade Federal de Minas Gerais (UFMG), Faculdade de Medicina, Departamento de Fonoaudiologia, Belo Horizonte, MG, Brazil

**Keywords:** Vestibular nerve, Motor evoked potential, Labyrinth vestibule, Vestibular disorders, Saccule and utricle, Nervo vestibular, Potencial evocado motor, Vestíbulo do labirinto, Doenças vestibulares, Sáculo e utrículo

## Abstract

**Introduction:**

The vestibular evoked myogenic potential is a potential of mean latency that measures the muscle response to auditory stimulation. This potential can be generated from the contraction of the sternocleidomastoid muscle and also from the contraction of extraocular muscles in response to high-intensity sounds. This study presents a combined or simultaneous technique of cervical and ocular vestibular evoked myogenic potential in individuals with changes in the vestibular system, for use in otoneurologic diagnosis.

**Objective:**

To characterize the records and analyze the results of combined cervical and ocular VEMP in individuals with vestibular hyporeflexia and in those with Ménière's disease.

**Methods:**

The study included 120 subjects: 30 subjects with vestibular hyporeflexia, 30 with Ménière's disease, and 60 individuals with normal hearing. Data collection was performed by simultaneously recording the cervical and ocular vestibular evoked myogenic potential.

**Results:**

There were differences between the study groups (individuals with vestibular hyporeflexia and individuals with Ménière's disease) and the control group for most of wave parameters in combined cervical and ocular vestibular evoked myogenic potential. For cervical vestibular evoked myogenic potential, it was observed that the prolongation of latency of the P13 and N23 waves was the most frequent finding in the group with vestibular hyporeflexia and in the group with Ménière's disease. For ocular vestibular evoked myogenic potential, prolonged latency of N10 and P15 waves was the most frequent finding in the study groups.

**Conclusion:**

Combined cervical and ocular vestibular evoked myogenic potential presented relevant results for individuals with vestibular hyporeflexia and for those with Ménière's disease. There were differences between the study groups and the control group for most of the wave parameters in combined cervical and ocular vestibular evoked myogenic potential.

## Resumo

**Introdução:**

O potencial evocado miogênico vestibular é um potencial de média latência que avalia a resposta muscular decorrente de estimulação auditiva. Este potencial pode ser gerado a partir da contração do músculo esternocleidomastoideo e também a partir da contração de músculos extraoculares em resposta a sons de elevada intensidade. Este estudo apresenta uma técnica combinada ou simultânea de potencial evocado miogênico vestibular cervical e ocular em indivíduos com alterações no sistema vestibular para que esta possa ser utilizada no diagnóstico otoneurológico.

**Objetivo:**

Caracterizar o registro e analisar os resultados do potencial evocado miogênico vestibular cervical e ocular combinado em indivíduos com hiporreflexia vestibular e em indivíduos com doença de Ménière.

**Método:**

Participaram do estudo 120 indivíduos, 30 com hiporreflexia vestibular, 30 com doença de Ménière e 60 com audição dentro dos padrões de normalidade. A coleta de dados foi feita por meio do potencial evocado miogênico vestibular cervical e ocular registrados simultaneamente.

**Resultados:**

Houve diferença entre o grupo de estudo (indivíduos com hiporreflexia vestibular e indivíduos com doença de Ménière) e o grupo controle para a maioria dos parâmetros das ondas no potencial evocado miogênico vestibular cervical e ocular combinado. Para o potencial evocado miogênico vestibular cervical observou-se que o prolongamento da latência das ondas P13 e N23 foi a alteração mais encontrada no grupo de indivíduos com hiporreflexia vestibular e no grupo de indivíduos com doença de Ménière. Para o potencial evocado miogênico vestibular ocular o prolongamento da latência das ondas N10 e P15 foi a alteração mais encontrada no grupo de estudo.

**Conclusão:**

O potencial evocado miogênico vestibular cervical e ocular combinado apresentou resultados relevantes para os indivíduos com hiporreflexia vestibular e para os indivíduos com doença de Ménière. Houve diferença entre o grupo de estudo e o grupo controle para a maioria dos parâmetros das ondas no potencial evocado miogênico vestibular cervical e ocular combinado.

## Introduction

The vestibular evoked myogenic potential (VEMP) is formed by myogenic responses activated by sound stimulation through high-intensity sounds. The literature describes two types of VEMP: cervical and ocular.^1^, ^2^, ^3^

Cervical VEMP activates the saccular macula, the inferior vestibular nerve, and the descending vestibulospinal pathways, recorded by surface electromyography of the cervical muscles in the presence of muscle contraction.^1^, ^2^ Ocular VEMP activates the utricular macula, the superior vestibular nerve, and the ascending vestibular pathways, recorded by surface electromyography on the extraocular muscles in the presence of muscle contraction.^4^, ^5^

Although relatively old – it was discovered in the mid-1960s – VEMP is still little known, and it comprises a vast universe of possible research and applications.^1^, ^2^, ^3^

The significance of electrical responses, the neural circuit involved, and the behavior of these responses in normal individuals have already been well demonstrated.^6^ However, although the findings of this exam in different neurological and otoneurological disorders have been described, much remains to be clarified and studied.^3^, ^4^, ^7^

In studies conducted in patients with unilateral vestibular dysfunction, a high variability of responses for cervical and ocular VEMP has been described.^8^, ^9^, ^10^, ^11^, ^12^, ^13^, ^14^, ^15^ For individuals with superior vestibular neuritis, a lack of response to ocular VEMP and normal responses to cervical VEMP were observed. However, for individuals with inferior vestibular neuritis, a lack of response to cervical VEMP and normal responses to ocular VEMP were observed.^10^, ^11^, ^12^

For individuals with superior semicircular canal dehiscence syndrome, increased amplitudes were observed for both cervical and ocular VEMP. According to the literature, there is a significant correlation between the size of dehiscence and the amplitudes of ocular VEMPs.^13^, ^14^, ^15^

For individuals with vestibular schwannoma, a prolonged latency for cervical VEMP was observed that can be attributed to tumor compression of the vestibulospinal tract and the compression of the myelin sheath on the inferior vestibular nerve. Regarding ocular VEMP, reduced or absent responses were observed for most individuals with vestibular schwannoma.^16^

Researchers have been using VEMP in the assessment of patients with Ménière's disease.^17^, ^18^, ^19^, ^20^, ^21^ Given that the mechanism through which endolymphatic hydrops develops is controversial and that the etiopathogenesis of the disease is still in the field of scientific speculation, new clinical instruments are needed to aid the identification of saccular hydrops.^18^, ^19^, ^20^

In a study that used cervical and ocular VEMP to assess patients with Ménière's disease, the authors observed a higher number of absent responses in ocular VEMP when compared with cervical VEMP. For those authors, the justification for such an occurrence was probably due to the fact the utricle is more associated with hearing function at low frequencies than the saccule.^17^

They also noted that VEMP response varied according to the stage of Ménière's disease, whether acute or during the interval between attacks. In the acute phase, the amplitude of ocular VEMP (contralateral to the affected ear) showed increased responses, while the amplitude of cervical VEMP (ipsilateral to the affected ear) was attenuated.^17^

In a study that evaluated the otolith function in patients with Ménière's disease during the acute phase and during the interval between attacks, it was observed that the increase in N10 wave amplitude was higher in the acute phase. However, the increase in N10 wave amplitude was much higher in the contralateral side of the affected ear than in the ipsilateral side of the affected ear.^18^

In another study, the authors found a high incidence of altered cervical VEMP responses in asymptomatic ears of patients with Ménière's disease. For those authors, VEMP can be an aid in the staging and follow-up of Ménière's disease. They also observed a very small number of delays in P13 and N23 wave latencies.^19^

In contrast, another study found altered cervical VEMP responses in asymptomatic ears of patients with Ménière's disease, but found no significant differences between the latencies of P13 and N23 waves of affected and asymptomatic ears. In that same study, the authors observed a lack of response in the asymptomatic ear in 20% of cases. For those authors, this fact shows the value of evoked myogenic potentials in the diagnosis of occult sacculus hydrops without clinical manifestations.^20^

The present study was justified by the possibility of simultaneously assessing the ipsilateral descending and contralateral ascending vestibular pathways in individuals with otoneurological changes, contributing to the accuracy of the combined cervical and ocular VEMP technique, as well as to its current use in otoneurological assessment.

This study aimed to characterize the record and analyze the results of combined cervical and ocular VEMP in individuals with vestibular hyporeflexia and in those with Ménière's disease.

## Methods

The procedures of this study were approved by the Research Ethics Committee from Universidade Federal de Minas Gerais (UFMG), under CAAE Protocol N° 32505314.0.0000.5149, in accordance with Resolution 466/12 of the National Health Council (Conselho Nacional de Saude [CONEP]).

This was a descriptive study with qualitative and quantitative analysis. One hundred and twenty subjects, aged 18–59 years, were invited to participate.

The sample comprised a study group of 30 individuals with vestibular hyporeflexia and 30 individuals with unilateral Ménière's disease, and an age- and sex-matched control group of 60 individuals without a diagnosis of peripheral disorders of the inner ear. The control group was subdivided into two groups of 30 individuals each. Control group 1 (CG1) was paired with the group of individuals with vestibular hyporeflexia and control group 2 (CG2) was paired with the group of patients with Ménière's disease.

The participants were selected at the UFMG School of Medicine and at the Audiology Clinic at the Sao Geraldo Annex from the University Hospital at UFMG and in Diagnostic Center Otorhinolaryngological through a non-random convenience sampling technique. The participants were notified personally of the research objectives, the absence of damage to their health, the assurance of secrecy of their identities or any other characteristics that could identify them, and about the research methods. After the necessary clarifications, all participants signed an informed consent.

Data collection was conducted at the Audiology Clinic at the São Geraldo Anexx from the University Hospital at UFMG and in Diagnostic Center Otorhinolaryngological. The individuals in the study group underwent otorhinolaryngologic evaluation, and all subjects (study group and control group) underwent a basic audiological evaluation. This evaluation consisted of: medical history, meatoscopy, pure tone audiometry, speech audiometry, tympanometry, and acoustic reflexes assessment.

For the medical history, the participant provided information such as personal data, audiological history, and aspects related to health. A Heine^®^ Mini 2000 otoscope was used for meatoscopy. Pure tone audiometry and speech audiometry were performed in a soundproof booth with a one-channel audiometer, Interacoustics^®^ AD 229b model, using TDH-39 earphones and a B-71 bone vibrator. Tympanometry and acoustic reflex assessment were performed using an Interacoustics^®^ AZ7 middle ear analyzer.

For the study group, inclusion criteria comprised individuals with vestibular hyporeflexia defined by vestibular assessment (electronystagmography or vector electronystagmography) and another group was composed by individuals diagnosed with Ménière's disease according to the Bárány Society criteria. The Bárány Society defines the following clinical criteria for the diagnose of Ménière's disease: two or more spontaneous episodes of vertigo lasting from 20 min to 12 h; sensorineural hearing loss (affecting mainly the middle frequencies) in the affected ear on at least one occasion before, during, or after one of the episodes of vertigo; presence of intermittent auditory symptoms such as hearing loss, ear fullness, and tinnitus on the affected side; and signs and symptoms that are not explained by another vestibular diagnosis.^22^

For the control group, inclusion criteria comprised individuals with no hearing complaints, no history of vestibular and/or otologic disease, and audiologic evaluation within normal standards. An audiologic evaluation was considered to be within normal limits when the pure tone air-conduction thresholds were 25 dB HL or less in the frequencies of 250–8000 Hz; pure tone bone-conduction thresholds were 15 dB HL or less in the frequencies of 500–4000 Hz, and the differences between the thresholds for air and bone conduction were less than or equal to 10 dB; additional requirements included type A tympanometric curve, and presence of acoustic reflexes at 500, 1000, 2000, and 4000 Hz. For the evaluation of pure tone thresholds, the criteria established by Silman and Silverman were used^23^; for the tympanometric curve, the criteria established by Jerger were used.^24^

The exclusion criteria comprised participants who had neurological disorders, cancer, otitis, tympanic membrane perforation, those with history of craniocerebral trauma, previous otologic surgery, and individuals who were unable to perform cervical rotation and eye movements. Considering that Ménière's disease may present with vestibular hyporeflexia at the vestibular evaluation, individuals with suspicion and/or diagnosis of Ménière's disease were excluded from the vestibular hyporeflexia group.

After the basic audiological evaluation, participants were referred for electrophysiological evaluation through the vestibular evoked myogenic potential (VEMP).

The VEMP was performed in a comfortable and quiet environment, with Labat^®^ equipment, using two channels. The stimuli were presented through ER 3A insertion phones, with disposable foam eartips. Tone burst stimulus at an intensity of 120 dB nHL were used. In this study, a bandpass filter of 10–1500 Hz was used. To obtain each record, 100 stimuli were presented at a frequency of 500 Hz at a rate of four stimuli per second. The scan window was 50 ms. Each subject underwent at least two stimulations per side, to verify the replication of the potential. The impedance values were checked before each record; they had to be below 5 kΩ.

To perform the VEMP, the participant's skin was cleaned with dehydrated alcohol followed by abrasive paste; surface electrodes received a small amount of electrolyte paste and were fixed with adhesive tape. For recording, the active electrode (negative electrode) in channel 1 was placed approximately 1 cm below the lower eyelid, and the reference electrode (positive electrode) was placed at a distance of approximately 1 cm from the active electrode. The active electrode on channel 2 was placed on the opposite side to the channel 1, at the anterior border of the sternocleidomastoid muscle in its upper third, and the reference electrode was placed in the sternal notch region. The ground electrode was placed on the forehead (Fpz). Therefore, the positioning of the electrodes allowed for the simultaneous recording of ocular and cervical VEMP; channel 1 was used for recording ocular VEMP and channel 2, for cervical VEMP.

Upon examination, the participant was instructed to sit on the chair and keep the head rotated to the opposite side of the stimulated ear, causing contraction of the sternocleidomastoid muscle. At the same time, the participant was instructed to look at a stationary target located on the wall of the test room and then immediately to a fixed point located above the target, which formed a vertical viewing angle of approximately 30° above the horizontal plane formed by the participant's head. Afterwards, the contralateral cervical and ocular VEMPs were recorded using the same technique.

After data collection, data were tabulated and submitted to statistical analysis. Statistical analysis was performed using SPSS version 20.0. Initially, a descriptive analysis was performed, which included measures of central tendency (mean and median), dispersion (standard deviation), and position (maximum and minimum). The normality of the samples was assessed using the Kolmogorov–Smirnov and Shapiro–Wilk tests. In addition to the descriptive statistics, inferential statistics was performed through Student's *t*-test and the Mann–Whitney test for comparison of two independent samples, and through Student's *t*-test and Wilcoxon test for comparison of paired samples. The chi-squared test was used to compare the frequencies obtained by calculating the asymmetry index and to compare hearing loss with the results of combined cervical and ocular VEMP. The level of significance of 5% (*p* ≤ 0.05) was adopted. Results significant at the 10% level (*p* ≤ 0.10) were considered as having a trend toward statistical significance.

## Results

The mean age of the study population was 49.4 years (SD = 7.03) for the group of individuals with post-caloric nystagmus hyporeflexia; for the CG1, the mean age was 49.1 years (SD = 7.12). For the group of patients with Ménière's disease, the mean age was 46.2 years (SD = 8.66); for the CG2, the mean age was 46.1 years (SD = 8.53).

Descriptive analysis for the group of individuals with vestibular hyporeflexia and the CG1 are shown in Table 1. For cervical VEMP, it was observed that the mean latency values for both P13 and N23 waves were higher in the group of patients with vestibular hyporeflexia. For ocular VEMP, the mean latency values of N10 and P15 waves were higher in the group of individuals with vestibular hyporeflexia.

**Table 1:** Measures of central tendency, dispersion, and position for latency (ms) and amplitude (μV) for combined cervical and ocular VEMP for individuals with vestibular hyporeflexia and individuals in the control group (CG1).

Wave parameters	Hyporeflexia					CG1				
	Mean	Median	SD	Max.	Min.	Mean	Median	SD	Max.	Min.
**St. RE**										
*Cervical*										
Ampl P13	26.16	22.72	19.03	74.47	0.00	34.48	32.63	18.16	74.22	7.64
Ampl N23	29.80	28.41	18.29	67.76	0.00	57.20	43.32	39.27	169.58	10.58
Inter ampl	56.07	55.97	33.57	128.73	0.00	91.67	76.51	54.38	225.27	18.22
Lat P13	15.94	14.60	5.70	31.30	0.00	12.82	12.90	1.07	14.90	10.70
Lat N23	23.33	22.05	6.18	37.80	0.00	22.19	21.90	1.46	24.90	20.50

*Ocular*										
Ampl N10	2.80	1.52	3.80	15.81	0.09	2.66	2.04	1.32	5.87	1.02
Ampl P15	2.64	1.67	3.31	12.95	0.10	3.34	2.60	1.98	8.82	1.22
Inter ampl	5.44	3.28	7.04	28.76	0.19	6.03	5.26	3.10	14.69	2.90
Lat N10	12.37	11.20	3.17	20.20	8.30	10.26	10.25	0.79	12.00	8.70
Lat P15	19.48	18.35	4.28	28.50	13.40	15.24	15.10	0.89	17.03	13.40

**St. LE**										
*Cervical*										
Ampl P13	19.34	15.33	13.59	51.41	0.00	26.53	22.89	13.37	66.69	9.08
Ampl N23	18.66	13.58	13.34	47.07	0.00	44.07	37.26	21.66	96.86	17.31
Inter ampl	38.00	34.49	25.26	91.03	0.00	70.60	62.41	32.47	163.55	28.17
Lat P13	17.50	16.45	5.92	28.30	0.00	12.99	12.75	1.05	15.10	10.90
Lat N23	25.41	25.70	6.87	35.40	0.00	22.57	22.35	1.49	25.30	20.10

*Ocular*										
Ampl N10	1.91	0.86	2.67	12.19	0.00	2.25	2.18	0.92	4.02	1.04
Ampl P15	1.92	0.68	2.56	10.60	0.00	2.55	2.68	0.83	4.09	1.12
Inter ampl	3.83	1.53	5.03	19.20	0.00	4.80	5.02	1.64	7.70	2.54
Lat N10	12.94	12.80	5.51	28.30	0.00	10.52	10.50	0.89	11.90	8.80
Lat P15	19.67	20.35	7.46	37.10	0.00	15.41	15.50	0.78	17.00	13.80

SD, standard deviation; Max., maximum, Min., minimum; St, stimulation; RE, right ear; LE, left ear; Ampl, amplitude; Lat, latency.

Descriptive analysis for the group of patients with Ménière's disease and for the CG2 is shown in Table 2. For cervical VEMP, it was observed that the mean latency values for both P13 and N23 waves were higher in the group of patients with Ménière's disease only with the right ear stimulation. For ocular VEMP, the mean latency values of N10 and P15 waves were higher in the group of patients with Ménière's disease for both the right ear and the left ear stimulation.

**Table 2:** Measures of central tendency, dispersion, and position for latency (ms) and amplitude (μV) for combined cervical and ocular VEMP for individuals with Ménière's disease and individuals in the control group (CG2).

Wave parameters	Ménière's disease					CG2				
	Mean	Median	SD	Max.	Min.	Mean	Med.	SD	Max.	Min.
**St. RE**										
*Cervical*										
Ampl P13	25.96	24.54	13.18	52.02	4.31	43.68	45.02	19.06	77.98	7.64
Ampl N23	34.59	31.42	20.25	85.63	6.90	62.28	60.49	27.16	111.38	10.58
Inter ampl	60.55	54.11	31.96	128.65	11.39	105.98	106.98	44.51	170.63	18.22
Lat P13	15.08	13.90	3.12	23.50	11.60	13.40	13.45	0.96	14.90	11.60
Lat N23	23.53	22.80	3.15	31.70	20.50	22.39	22.45	1.48	24.90	20.00

*Ocular*										
Ampl N10	4.40	1.87	7.63	28.81	0.14	2.33	2.05	0.80	5.25	1.04
Ampl P15	5.95	1.89	12.32	45.18	0.15	3.21	3.05	1.18	8.48	1.13
Inter ampl	10.35	3.88	19.93	73.70	0.29	5.57	5.26	1.90	13.73	2.17
Lat N10	12.18	10.95	3.49	22.60	9.10	10.40	10.40	0.84	12.10	9.20
Lat P15	18.13	16.45	3.94	28.50	13.80	15.27	15.05	0.96	17.20	13.40

**St. LE**										
*Cervical*										
Ampl P13	23.73	21.98	13.77	54.56	0.00	37.64	32.47	15.63	76.04	14.97
Ampl N23	29.42	29.60	16.19	68.61	0.00	51.47	51.34	16.88	95.88	23.74
Inter ampl	53.15	50.75	28.91	123.17	0.00	89.11	83.84	29.77	158.22	39.60
Lat P13	14.05	13.40	4.82	22.70	0.00	13.18	13.30	1.16	14.90	11.20
Lat N23	21.40	20.95	6.47	29.30	0.00	22.65	22.60	1.36	24.80	20.50

*Ocular*										
Ampl N10	1.58	1.55	0.88	3.37	0.00	2.64	2.50	0.96	5.34	1.07
Ampl P15	1.72	1.79	1.11	4.76	0.00	3.29	3.02	1.31	8.29	1.12
Inter ampl	3.30	3.61	1.91	7.95	0.00	5.93	5.53	2.21	13.63	2.53
Lat N10	11.95	11.05	4.42	28.30	0.00	10.49	10.30	0.94	12.20	9.10
Lat P15	17.42	16.80	5.62	37.10	0.00	15.38	15.25	0.83	17.10	13.80

SD, standard deviation; Max., maximum, Min., minimum; St, stimulation; RE, right ear; LE, left ear; Ampl, amplitude; Lat, latency.

In the inferential statistical analysis for the group of subjects with vestibular hyporeflexia and the CG1, a difference was observed between the groups for most wave parameters (Table 3). For the group of patients with Ménière's disease and the CG2, differences between the groups for most wave parameters were also observed (Table 4).

**Table 3:** Comparison of individuals with vestibular hyporeflexia and the control group (CG1) for latency (ms) and amplitude (μV) for combined cervical and ocular VEMP.

Parameters	Hyporeflexia			CG1			*p*-value
	Mean	Median	SD	Mean	Median	SD	
**Stimulation RE**							
*Cervical*							
Ampl P13	26.16	22.72	19.03	34.48	32.63	18.16	0.090^a^^,^^c^
Ampl N23	29.80	28.41	18.29	57.20	43.32	39.27	**0.001**^b^
Inter ampl	56.07	55.97	33.57	91.67	76.51	54.38	**0.005**^b^
Lat P13	15.94	14.60	5.70	12.82	12.90	1.07	**0.006**^b^
Lat N23	23.33	22.05	6.18	22.19	21.90	1.46	0.151^a^

*Ocular*							
Ampl N10	2.80	1.52	3.80	2.66	2.04	1.32	0.088^a^^,^^c^
Ampl P15	2.64	1.67	3.31	3.34	2.60	1.98	**0.026**^a^
Inter ampl	5.44	3.28	7.04	6.03	5.26	3.10	**0.024**^b^
Lat N10	12.37	11.20	3.17	10.26	10.25	0.79	**0.001**^b^
Lat P15	19.48	18.35	4.28	15.24	15.10	0.89	**<0.001**^b^

**Stimulation LE**							
*Cervical*							
Ampl P13	19.34	15.33	13.59	26.53	22.89	13.37	**0.045**^b^
Ampl N23	18.66	13.58	13.34	44.07	37.26	21.66	**<0.001**^b^
Inter ampl	38.00	34.49	25.26	70.60	62.41	32.47	**<0.001**^b^
Lat P13	17.50	16.45	5.92	12.99	12.75	1.05	**<0.001**^b^
Lat N23	25.41	25.70	6.87	22.57	22.35	1.49	**0.033**^b^

*Ocular*							
Ampl N10	1.91	0.86	2.67	2.25	2.18	0.92	**0.038**^a^
Ampl P15	1.92	0.68	2.56	2.55	2.68	0.83	0.054^a^^,^^c^
Inter ampl	3.83	1.53	5.03	4.80	5.02	1.64	**0.035**^a^
Lat N10	12.94	12.80	5.51	10.52	10.50	0.89	**0.028**^b^
Lat P15	19.67	20.35	7.46	15.41	15.50	0.78	**0.004**^b^

SD, standard deviation; RE, right ear; LE, left ear; Ampl, amplitude; Lat, latency.

Bold values signify (*p* ≤ 0.05).

a Wilcoxon's test (*p* ≤ 0.05).

b *t*-test (*p* ≤ 0.05).

c Values with trend toward statistical significance (*p* ≤ 0.10)

**Table 4:** Comparison of individuals with Ménière's disease and the control group for latency (ms) and amplitude (μV) for combined cervical and ocular VEMP.

Parameters	Ménière's disease			CG2			*p*-value
	Mean	Median	SD	Mean	Median	SD	
**Stimulation RE**							
*Cervical*							
Ampl P13	25.96	24.54	13.18	43.68	45.02	19.06	**<0.001**^b^
Ampl N23	34.59	31.42	20.25	62.28	60.49	27.16	**<0.001**^b^
Inter ampl	60.55	54.11	31.96	105.98	106.98	44.51	**<0.001**^b^
Lat P13	15.08	13.90	3.12	13.40	13.45	0.96	**0.010**^b^
Lat N23	23.53	22.80	3.15	22.39	22.45	1.48	0.083^b^^,^^c^

*Ocular*							
Ampl N10	4.40	1.87	7.63	2.55	1.94	1.29	0.713^a^
Ampl P15	5.95	1.89	12.32	3.81	3.45	2.04	**0.018**^a^
Inter ampl	10.35	3.88	19.93	6.36	5.39	3.23	0.063^a^^,^^c^
Lat N10	12.18	10.95	3.49	10.05	10.10	0.56	**0.038**^a^
Lat P15	18.13	16.45	3.94	14.61	14.60	0.59	**0.001**^b^

**Stimulation LE**							
*Cervical*							
Ampl P13	23.73	21.98	13.77	37.64	32.47	15.63	**<0.001**^b^
Ampl N23	29.42	29.60	16.19	51.47	51.34	16.88	**<0.001**^b^
Inter ampl	53.15	50.75	28.91	89.11	83.84	29.77	**<0.001**^b^
Lat P13	14.05	13.40	4.82	13.18	13.30	1.16	**0.043**^a^
Lat N23	21.40	20.95	6.47	22.65	22.60	1.36	0.316^b^

*Ocular*							
Ampl N10	1.58	1.55	0.88	2.64	2.50	0.96	**<0.001**^b^
Ampl P15	1.72	1.79	1.11	3.29	3.02	1.31	**<0.001**^b^
Inter ampl	3.30	3.61	1.91	5.93	5.53	2.21	**<0.001**^b^
Lat N10	11.95	11.05	4.42	10.49	10.30	0.94	**0.047**^a^
Lat P15	17.42	16.80	5.62	15.38	15.25	0.83	**0.002**^a^

SD, standard deviation; RE, right ear; LE, left ear; Ampl, amplitude; Lat, latency.

Bold values signify (*p* ≤ 0.05).

a Wilcoxon's test (*p* ≤ 0.05).

b *t*-test (*p* ≤ 0.05).

c Values with trend toward statistical significance (*p* ≤ 0.10).

When comparing the right and left ears in both the group with vestibular hyporeflexia and in the group with Ménière's disease, no differences between the ears in cervical and ocular VEMP were observed.

Regarding the asymmetry index, no difference between the study and control groups were observed at cervical VEMP. The asymmetry index ranged from 0% to 88%.

**Figure 1:**
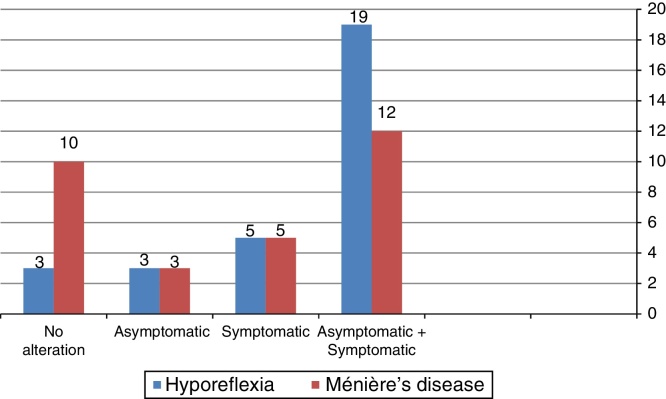
Frequency distribution of the outcome of combined cervical and ocular VEMP, per the affected side, for individuals with vestibular hyporeflexia and for individuals with Ménière's disease (*n* = 30).

Fig. 1 shows that the combined cervical and ocular VEMP was abnormal in asymptomatic ears of the individuals in the group with vestibular hyporeflexia and in the group with Ménière's disease.

**Figure 2:**
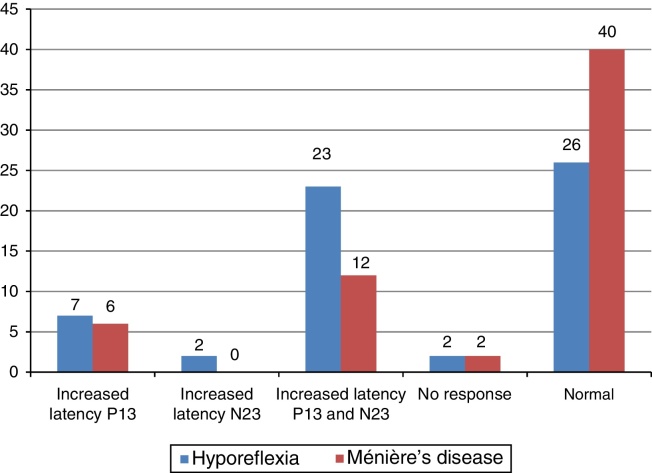
Frequency distribution of the results of cervical VEMP, per ear, for individuals with vestibular hyporeflexia and for individuals with Ménière's disease (*n* = 60).

**Figure 3:**
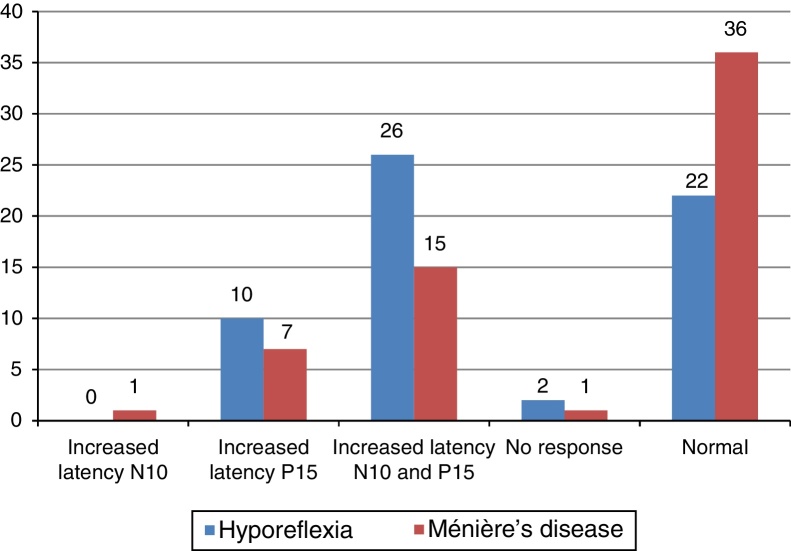
Frequency distribution of the results of ocular VEMP, per ear, for individuals with vestibular hyporeflexia and for individuals with Ménière's disease (*n* = 60).

The main alteration observed at cervical VEMP in the group with vestibular hyporeflexia and in the group with Ménière's disease was the prolonged P13 and N23 wave latency (Fig. 2). The main alteration observed at ocular VEMP in the group with vestibular hyporeflexia and in the group with Ménière's disease was also the prolonged P13 and N23 wave latency (Fig. 3).

It is noteworthy that 13 (43.30%) individuals from the vestibular hyporeflexia group had alterations in both the cervical and ocular VEMP. In the group with Ménière's disease, six (20%) patients had changes in both cervical and ocular VEMP.

Regarding the side of the hearing loss, of the 30 individuals with vestibular hyporeflexia, seven (23.35%) had unilateral hearing loss, seven (23.35%) had bilateral hearing loss, and 16 (53.30%) had bilateral normal hearing. Of the 30 patients with Ménière's disease, 11 (36.70%) had unilateral hearing loss, 13 (43.30%) had bilateral hearing loss, and six (20%) had bilateral normal hearing. It is noteworthy that hearing loss was sensorineural in both individuals with vestibular hyporeflexia and individuals with Ménière's disease.

**Figure 4:**
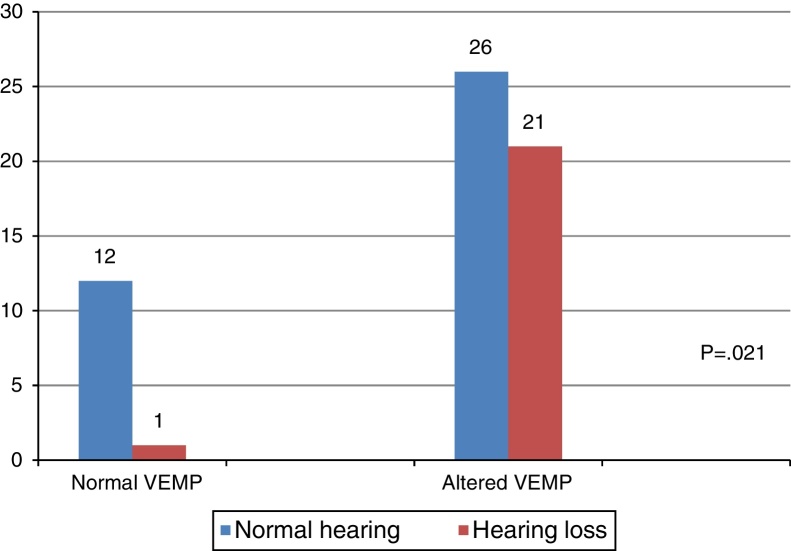
Comparative analysis of the results of combined cervical and ocular VEMP and hearing, per ear, for individuals with vestibular hyporeflexia (*n* = 60). Chi-squared test (*p* ≤ 0.05) or Fisher's exact test (*p* ≤ 0.05).

When comparing the results of the combined cervical and ocular VEMP with respect to hearing, it was observed that individuals with vestibular hyporeflexia presented a difference in the results of combined cervical and ocular VEMP between the ears with normal hearing and those with hearing loss (Fig. 4).

**Figure 5:**
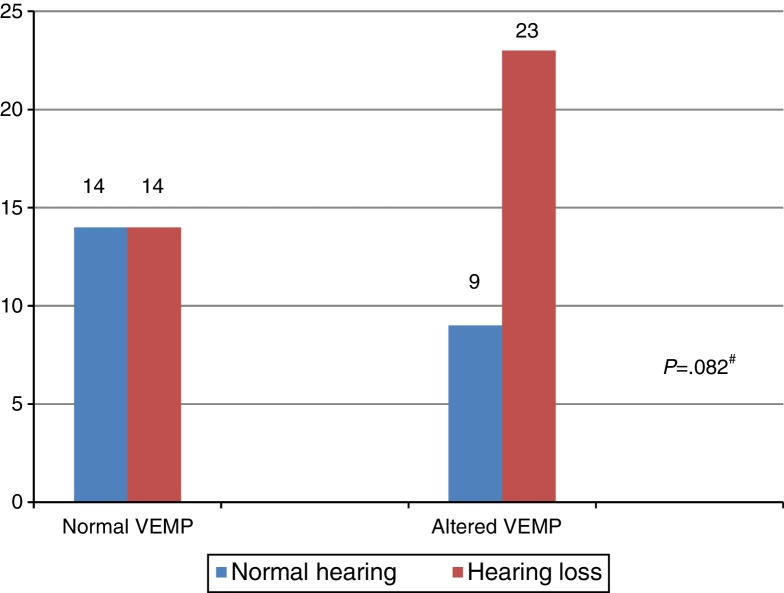
Comparative analysis of the results of combined cervical and ocular VEMP and hearing, per ear, for individuals with Ménière's disease (*n* = 60). Chi-squared test (*p* ≤ 0.05) or Fisher's exact test (*p* ≤ 0.05). #Values with trend toward statistical significance (*p* ≤ 0.10).

In turn, the comparison of the combined cervical and ocular VEMP results regarding hearing in individuals with Ménière's disease demonstrated that there was a tendency toward a difference in the results of combined cervical and ocular VEMP between the ears with normal hearing and those with hearing loss (Fig. 5).

In the comparative analysis of the results of combined cervical and ocular VEMP and the degree of hearing loss, Table 5 shows that, for individuals with vestibular hyporeflexia, there was a higher frequency of changes in the degree of mild hearing loss. For individuals with Ménière's disease, there was a tendency for a higher frequency of changes in the degree of moderate hearing loss (Table 5).

**Table 5:** Comparison of the degree of hearing loss, per ear, in vestibular hyporeflexia and Ménière's disease in relation to the outcome of combined cervical and ocular VEMP (*n* = 60).

	VEMP results				
	Normal	Altered	*p*-value	Odds ratio	95% CI
	*n* (%)	*n* (%)			
**Hyporeflexia**					
*Hearing loss*					
Absent	12 (20.00)	26 (43.30)	–	–	–
Mild	0 (0.00)	12 (20.00)	**0.047**	0.684	0.55–0.85
Moderate	0 (0.00)	6 (10.00)	0.167	–	–
Moderately severe	1 (1.70)	1 (1.70)	1.000	–	–
Severe	0 (0.00)	2 (3.30)	1.000	–	–

**Ménière's disease**					
*Hearing loss*					
Absent	14 (23.30)	9 (15.00)	–	–	–
Mild	7 (11.70)	11 (18.30)	0.162	–	–
Moderate	4 (6.70)	11 (18.30)	0,052^a^	4.278	1.04–17.66
Severe	1 (1.70)	2 (3.30)	0.556	–	–
Deep	1 (1.70)	0 (0.00)	1.000	–	–

CI, confidence interval.

Bold values signify (*p* ≤ 0.05).

Chi-squared test (*p* ≤ 0.05) or Fisher's exact test (*p* ≤ 0.05).

a Values with a tendency to statistical significance (*p* ≤ 0.10).

When comparing the results of cervical and ocular VEMP, no differences were observed for the frequency of normal and altered responses in the group with vestibular hyporeflexia and in the group with Ménière's disease.

It is noteworthy that in the group with vestibular hyporeflexia, 34 (57%) ears showed abnormalities in cervical VEMP and 38 (63%) ears presented change in ocular VEMP. In the group of patients with Ménière's disease, 20 (33%) ears showed abnormalities in cervical VEMP and 24 (40%) ears had alterations in ocular VEMP.

## Discussion

The importance of VEMP is related to the functional assessment of the pathways involved in conducting the stimulus from the inner ear to the reflex muscle response. The advantage of this test is that alterations yet undetected, or those that are not visible by imaging tests, can be detected early by VEMP.^3^ The analysis of the responses of the combined cervical and ocular VEMP showed satisfactory results for complementing the diagnostic assessment of individuals with vestibular hyporeflexia and those with Ménière's disease.

Since all subjects in the control groups showed normal responses to both cervical and ocular VEMP, this indicated the integrity of the saccular and utricular macula, inferior and superior vestibular nerve, vestibular nuclei, vestibular pathways, and effector muscle. Therefore, this assumption can explain the difference found, for the majority of wave parameters, between the group with vestibular hyporeflexia and CG1, as well as the difference observed (also for most wave parameters) between the group with Ménière's disease and CG2.

In the comparison between the right and left ears in both the group with vestibular hyporeflexia and in the group with Ménière's disease, there was no difference between the ears. However, the asymptomatic ears of individuals in the study group showed alterations in the combined cervical and ocular VEMP response.

In a study of patients with Ménière's disease, the authors observed prolonged P13 wave latency in asymptomatic ears of 15% of subjects. For those authors, the high endolymphatic pressure that hinders the transmission of sound would cause the prolonged P13 wave latency in asymptomatic ears, provided that the hearing in the non-affected side was impaired.^20^

In the present study, it was observed that seven (23%) individuals from the group with Ménière's disease who had prolonged P13 wave latency in the asymptomatic ear had hearing loss in the unaffected side. For some authors, prolonged P13 wave latency suggests retro-labyrinthine injury.^21^

For individuals with vestibular hyporeflexia, the cervical and ocular VEMP responses vary according to the type of otoneurological disease affecting the vestibular system. The literature reports alterations in asymptomatic ears for individuals with vestibular neuritis and for those with superior semicircular canal dehiscence syndrome.^8^, ^9^, ^10^, ^11^, ^12^, ^13^, ^14^ Alterations in asymptomatic ears were not observed in individuals with vestibular schwannoma.^16^

VEMP provides information that may be useful in the assessment of saccule and utricle involvement in several otoneurological diseases, both in the affected and in the asymptomatic ear. Otoneurological diseases involve the various labyrinthine segments in different ways, which explains the heterogeneity of response in diseases with unilateral involvement.^8^, ^9^, ^19^, ^20^, ^21^

The alterations observed in combined cervical and ocular VEMP in the asymptomatic ears of the study group may also be explained by the fact that VEMP assesses not only the neural structures, but mainly the sensory structures of the saccule and utricle, which are sensitive and responsive to acoustic stimulus, despite not contributing to the hearing capacity.^8^, ^19^

Regarding the asymmetry index, it was observed that, for both the group with vestibular hyporeflexia and the group with Ménière's disease, the value ranged from 0% to 88%. The literature describes normal values as those up to 47%.^15^ The variability of the responses is due to individual differences in the degree of contraction, tone, and mass of the studied muscle, despite the standardization of the individual posture during the performance of cervical VEMP.^1^, ^2^, ^3^

The increase in the asymmetry of amplitude of potentials index suggests hypersensitivity of saccular macula. In Ménière's disease, this increase indicates sacculus hydrops.^19^, ^20^, ^21^ In the present study, two (7%) individuals in the group with Ménière's disease showed an increase in asymmetry index. Conversely, in two (7%) individuals of the same group, an absence of ipsilateral or contralateral responses on the affected side was observed, which suggests saccular macula areflexia, and therefore a more advanced stage of the disease in this organ.^19^, ^20^, ^21^

In the group of patients with vestibular hyporeflexia, it was observed that seven (23%) patients showed an increase in asymmetry index and two (7%) subjects had no ipsilateral or contralateral responses on the affected side.

The integrity of the sacculo-collic reflex is confirmed by the presence of biphasic P13-N23 wave at cervical VEMP.^17^, ^19^, ^20^ This was observed in the affected ear of most individuals with vestibular hyporeflexia and in most individuals with Ménière's disease. However, the biphasic P13-N23 wave presented with increased latency values (whether only for the P13 wave, for the N23 wave, or for both) in 17 (57%) involved ears in the group with vestibular hyporeflexia and in ten (33%) involved ears in the group with Ménière's disease.

The lack of cervical VEMP response is attributed to insufficient muscle contraction during the assessment, hidden peripheral vestibular disorder, or hyposensitivity of the saccule due to saccular macula aging in the elderly.^1^, ^2^, ^3^, ^9^ In the present study, cervical VEMP was absent in one patient (3%) in the group with vestibular hyporeflexia and in one (3%) in the group with Ménière's disease.

The absence of cervical VEMP response in cases of Ménière's disease suggests saccular hydrops. Depending on the degree of severity of hydrops, some individuals may present an irreversible degeneration of the sensory epithelium of the saccular macula.^17^, ^19^, ^20^, ^21^ The lack of response in the non-affected ear was observed in 3% of individuals, both in the group with vestibular hyporeflexia and in the group with Ménière's disease. These findings may highlight the value of cervical VEMP in the diagnosis of occult sacculus hydrops without clinical manifestations.

The integrity of the utricular reflex is confirmed by the presence of a biphasic N10-P15 wave at ocular VEMP and depends on the stimulus mode (air or bone conduction) and on the action of the inferior oblique muscles involved in eye movement.^4^, ^5^ The biphasic N10-P15 wave occurred in the affected ear of most individuals with vestibular hyporeflexia and Ménière's disease. However, the biphasic N10-P15 wave had increased latency values (whether only for the N10 wave, for the P15 wave, or for both) in 18 (60%) affected ears in the group with vestibular hyporeflexia and in 11 (37%) affected ears in the group with Ménière's disease.

Ocular VEMP represents the path of the vestibular-ocular reflex. When a missing or asymmetrical reflex is found, injuries at any point along the route need to be considered. Delayed reflexes are typically observed in central nervous system diseases. The literature describes a variety of results for ocular VEMP. The responses vary according to the disease affecting the vestibular system (central or peripheral), the stage of the disease, the stimulus used, and the intensity and duration of the stimulus.^4^, ^5^, ^6^ Therefore, it becomes difficult to draw comparisons, since studies with similar methodologies were not retrieved.

Although there were no differences in the frequency of normal and altered responses in the cervical and ocular VEMP, more alterations were observed in the latter when compared with the former both in the group with vestibular hyporeflexia and the group with Ménière's disease. This fact is consistent with the literature and suggests that the utricular function may be more related to the auditory function at low frequencies than to the saccular function.^1^, ^2^, ^4^, ^5^, ^17^

VEMP relies solely on the integrity of the vestibular system, which allows it to be measured in individuals with hearing loss.^1^, ^2^, ^3^ In the present study, there was a difference between the results of combined cervical and ocular VEMP in relation to hearing, both for the individuals in the group with vestibular hyporeflexia and the group with Ménière's disease.

VEMP is not influenced by the hearing level of the subject evaluated. However, the increase in the degree of hearing loss may suggest a greater involvement of the organs of the inner ear, including the saccule and utricle.^1^, ^2^, ^4^ In the present study, there was a higher frequency of alterations in the combined cervical and ocular VEMP in the degree of mild hearing loss of individuals with vestibular hyporeflexia. For individuals with Ménière's disease, alterations were more frequently observed with a moderate degree of hearing loss. Therefore, these results should be considered with caution, since the data is not consistent with the literature.^1^, ^2^, ^4^

The literature describes that, in the air conduction stimulation, alterations in the middle ear cause changes in the record regarding the increased latency of this potential. However, the middle ear condition would have no significant effect on the VEMP record in bone conduction stimulus.^6^

It is noteworthy that air conduction stimulus was used in this study. Nonetheless, the sample for the control and study groups did not include individuals with middle ear disorders. Therefore, the results were not influenced by a middle ear disorder.

The combined cervical and ocular VEMP showed different results for the studied groups. This diversity results from different pathophysiologic disease processes. The differences can provide information about which receptors and/or pathways present dysfunction. However, further studies with similar methodology and involving more diverse otoneurological diseases should be performed.

The morbidity rates of various otoneurological diseases associated with late diagnosis justify the development of increasingly accurate methods for their diagnosis. Given that vestibular schwannoma affects approximately two people per 100,000, that vestibular neuritis is responsible for 15% of causes of vertigo, and that Ménière's disease affects approximately 43 people per 100,000, combined cervical and ocular VEMP emerges as a method in the diagnosis and monitoring of otoneurological diseases.^11^, ^16^, ^21^

It is important to note that, as with any other evoked potential, there is no specific correlation between alterations and disease, because many of the abnormalities found are similar for several diseases. Conversely, VEMP has several advantages to be considered: it is easy to perform and interpret, it is non-invasive, and it is not uncomfortable for the patient. Thus, this method deserves to be included in routine clinical otoneurological assessment.

## Conclusion

Combined cervical and ocular VEMP presented relevant results for individuals with vestibular hyporeflexia and for those with Ménière's disease. There were differences between the study groups and the control groups for most of the wave parameters in combined cervical and ocular VEMP.

## Conflicts of interest

The authors declare no conflicts of interest.
